# Perturbed states of the bacterial chromosome: a thymineless death case study

**DOI:** 10.3389/fmicb.2015.00363

**Published:** 2015-04-24

**Authors:** Lev Ostrer, Bree L. Hamann, Arkady Khodursky

**Affiliations:** Department of Biochemistry, Molecular Biology and Biophysics, Biotechnology Institute, University of Minnesota, St. Paul, MN, USA

**Keywords:** transcription, replication, spatial correlations, thymine starvation, thymineless death

## Abstract

Spatial patterns of transcriptional activity in the living genome of *Escherichia coli* represent one of the more peculiar aspects of the *E. coli* chromosome biology. Spatial transcriptional correlations can be observed throughout the chromosome, and their formation depends on the state of replication in the cell. The condition of thymine starvation leading to thymineless death (TLD) is at the “cross-roads” of replication and transcription. According to a current view, e.g., (Cagliero et al., 2014), one of the cellular objectives is to segregate the processes of transcription and replication in time and space. An ultimate segregation would take place when one process is inhibited and another is not, as it happens during thymine starvation, which results in numerous molecular and physiological abnormalities associated with TLD. One of such abnormalities is the loss of spatial correlations in the vicinity of the origin of replication. We review the transcriptional consequences of replication inhibition by thymine starvation in a context of the state of DNA template in the starved cells and opine about a possible significance of normal physiological coupling between the processes of replication and transcription.

## Introduction

Replication and transcription are intertwined in a number of ways, as molecular reactions and biological processes. In the cell, the two polymerization reactions utilize, and compete for, the same DNA template. That may force local interactions between DNA and RNA polymerase complexes (Liu and Alberts, 1995; Felipe-Abrio et al., 2014), which may be a part of the normal replication process (French, 1992; Merrikh et al., 2011) or, under special circumstances, may be a source of chromosome pathology (Merrikh et al., 2012). The reactions are also linked at a deep evolutionary level via the activity of the enzyme ribonucleoside di(tri)phosphate reductase which converts ribonucleotides, precursors of substrates (or substrates) for the reaction of transcription, into deoxyribonucleotides, substrates for DNA polymerization reaction (Reichard, 2010).

The biological processes of replication and transcription are also coupled via a number of regulatory mechanisms, some of which are still unexplained or/and whose physiological significance is not yet understood. First, DNA replication determines intrinsic levels of gene expression by establishing gene dosage gradients along the chromosome (Chandler and Pritchard, 1975; Schmid and Roth, 1987; Couturier and Rocha, 2006; Slager et al., 2014). Second, transcription is required for initiation of chromosomal DNA replication (Lark, 1972; Messer, 1972). Third, DNA replication is required for establishing normal spatial patterns of chromosomal transcription (Jeong et al., 2006). Fourth, interference with DNA replication results in a specialized DNA damage transcriptional response (Simmons et al., 2008; Kreuzer, 2013). Fifth, the replication initiator protein DnaA (Kaguni, 2006) controls, as an activator and repressor, transcription of the ribonucleotide reductase operon (Olliver et al., 2010). Sixth, activity of both processes can be modulated by (p)ppGpp (Chiaramello and Zyskind, 1990; Levine et al., 1991; Denapoli et al., 2013) and by changes in DNA supercoiling (Kowalski and Eddy, 1989; Crooke et al., 1991; von Freiesleben and Rasmussen, 1992; Peter et al., 2004; Rovinskiy et al., 2012), two molecular sensors that can relay information about critical fluctuations in the cell’s environment to the parts of the replication and transcription machineries (Dorman, 2006; Jin et al., 2012; Sobetzko et al., 2012). Last and perhaps more of a fortuitous link is a genetic one, exemplified by the evolutionarily conserved arrangement of genes encoding the DNA primase DnaG and the main sigma factor RpoD in one operon (Burton et al., 1983; Versalovic et al., 1993).

All these interactions have been shaped and calibrated by the evolutionary forces to accommodate species specific differences in genome organization and physiology. Disruption of normal temporal and spatial relationships between replication and transcription may have detrimental consequences for the cell. And nowhere such consequences are more pronounced than during thymine starvation.

## Thymine Starvation and Transcription

Thymine is one of the five common nucleobases and it is found primarily in DNA where it makes a Watson-Crick pair with Adenine. Active thymine compound in the cell is TTP: it is a precursor of DNA as well as of thymidine nucleotide sugars that serve as intermediates in O-antigen biosynthesis (Samuel and Reeves, 2003). In most organisms, cells synthesize thymine on the level of a nucleotide as thymidine monophosphate from deoxyuridine monophosphate (www.kegg.jp, www.ecocyc.org). Thymine dependency, or auxotrophy, can be established by mutating a gene encoding thymidylate synthase (Belfort et al., 1983). Thymine requiring mutants can only grow in presence of the exogenous base or nucleoside (Barner and Cohen, 1954). Starving cells for thymine results in a rapid decay in colony counts (Barner and Cohen, 1954). The decay is preceded by a near complete cessation of DNA synthesis (Barner and Cohen, 1958; Kuong and Kuzminov, 2010) and it is paralleled by increase in the cell size (Barner and Cohen, 1954), which is indicative of division arrest. However, inhibition of RNA and protein synthesis across a population of starved cells lags significantly behind the drop in colony formation (Barner and Cohen, 1958; McFall and Magasanik, 1960). Thus, cells deprived of thymine go through temporally ordered events: 1-inhibition of bulk DNA synthesis; 2-cell elongation and division arrest; 3-decrease in colony formation; 4-inhibition of protein and RNA synthesis. The overall phenomenon of thymine starvation leading to an exponential decrease in colony counts is known as thymineless death (Cohen and Barner, 1954), or TLD. It was postulated that the cause of death is “inhibition of DNA synthesis under conditions of continued cytoplasmic synthesis” (Barner and Cohen, 1957), including RNA, protein and overall biomass. Such uncoupling between biosynthetic processes was termed an “unbalanced growth” (Cohen and Barner, 1954) and it was proposed to be the underlying macro-mechanism of the bactericidal effect of not only thymine starvation, but also of a number of antibiotics (Barner and Cohen, 1956). However, the pathology of TLD has been associated with the transcriptional activity of the starved cells and not with other aspects of the unbalanced growth (Gallant and Suskind, 1962; Hanawalt, 1963; Rolfe, 1967; Morganroth and Hanawalt, 2006; Martin et al., 2014).

Transcriptional activity of a population of bacterial cells is a sum of the activities of individual cells. Even when growth rate of a population is kept constant, cells making up that population may have different microenvironments, may come from different stages of the cell cycle, and may be of different sizes and ages. As a result, transcriptional activity across a population of cells in a steady state can be viewed as a type of noise that must be deconvoluted on the basis of physiological parameters in order to make sense of the activity. In part because transcriptional activity is noisy, even under controlled conditions, transcriptional states of the cell have been traditionally characterized in terms of dominant differences in transcript abundances, or in promoter activities, which could be associated with physiological or/and environmental changes. Activation of the DNA damage sensitive promoter of the *recA* gene was originally shown to be one of such dominant changes elicited by thymine deprivation (Casaregola et al., 1982).

Introduction of whole-genome microarray technology (Schena et al., 1995) made monitoring transcriptional activity less biased and more quantitative. Genome-wide surveys of changes in transcript abundances elicited by thymine deprivation confirmed that the condition induces the SOS regulon (Sangurdekar et al., 2010, 2011), a collection of genetically unlinked genes whose transcription is primarily controlled by the LexA repressor and whose activities allow the cell to repair or bypass DNA lesions in time before the delayed cell division (Simmons et al., 2008). The surveys also found that activity of the SOS regulon contrasts thymine limitation and thymine deprivation: whereas both limitation and starvation result in transcriptional upregulation of genes of the deoxyribose salvage pathway and downregulation of genes whose products are involved in pyrimidine biosynthesis, only the starvation induces transcription of the SOS regulon (Sangurdekar et al., 2010, 2011).

Relative transcript abundances measured for nearly every gene in a genome can be used to correlate transcriptional activities of genes over time or conditions (Eisen et al., 1998; Tamayo et al., 1999).The resulting correlation patterns can be explained in terms of underlying regulatory mechanisms, e.g., (van Helden et al., 1998). Co-transcriptional patterns in prokaryotic genomes result primarily from organization of genes in operons (Gutierrez-Rios et al., 2003) and regulons (Khodursky et al., 2000a; Courcelle et al., 2001). However, an operonal organization can be inferred not only from multidimensional comparative profiles of transcriptional activity of genomes, but also from one dimensional spatial series of transcript abundances, strongly suggesting that transcriptional signals recorded across a population of cells, despite being a product of multiple sources of biological variation, contain biologically relevant structural information (Jeong et al., 2004). In fact, the spatial transcriptional signal can be used to model geometric features of the chromosomal DNA superstructure, assuming juxtaposition of co-regulated genes in 3-D space (Xiao et al., 2011). Mapping of contacts between linearly non-adjacent DNA segments in the *Caulobacter crescentus* chromosome using the high-resolution chromosome capture methodology (Lieberman-Aiden et al., 2009) revealed the general properties of physical organization of chromosomal DNA inside the cell (Le et al., 2013). These properties, including the dimensions of short- and medium-range features, were consistent with the estimates obtained from the geometric model which was based on expression data alone. This concordance bolsters the notion that spatial transcriptional profiles contain information about underlying structure of the chromosomal DNA inside the cell (Jeong et al., 2004).

**FIGURE 1:**
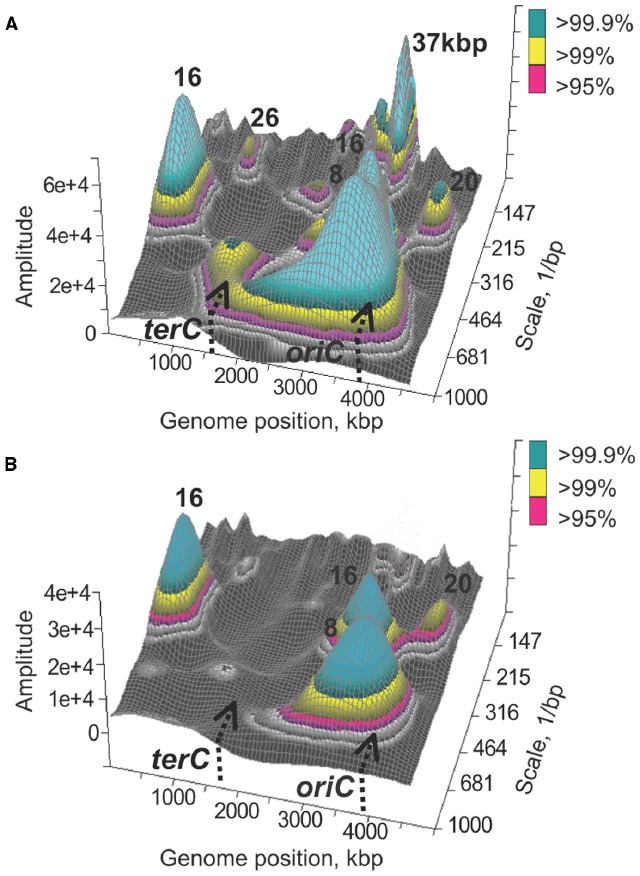
**Spatial correlations in transcript abundances along the *E. coli* chromosome.** Significant wavelets with corresponding main frequencies are shown for a genome-wide transcriptional signal recorded prior to thymine starvation **(A)** and 30 min into starvation **(B)**. The figure was obtained using publicly available data as previously described (Jeong et al., 2004).

Phenomenologically, transcriptional activity of any two genes can be up or down relative to some baseline level. If activities of multiple pairs of genes vary in the same direction, and genes in the pairs are situated the same or nearly the same distance apart, then the transcriptional signal can be viewed as spatially regular with a characteristic distance between genes with co-varying activity. Such characteristic distance, or spatial frequency, can be evaluated using conventional signal processing techniques, including autocorrelation or the Fourier transform. Using these methods, it was demonstrated that, independently of growth conditions, transcriptional signal from the *E. coli* chromosome can be characterized by several statistically significant spatial frequencies: 1 kbp, 4 ± 2 kbp, 16 ± 4 kbp, 32 ± 6 kbp, 128 ± 12 kbp, and 570 ± 50 kbp (Jeong et al., 2004). However, co-variations in transcriptional activities do not have to be spatially regular or have the same regularity across the entire chromosome. To account for that the signal can be analyzed using the wavelet transform which provides space-frequency representation of original spatial series (Torrence and Compo, 1998). Moreover, it may be useful to know what spectral components occur in which chromosomal intervals, especially for the purposes of comparative analysis (Allen et al., 2006). As expected, chromosome wide transcriptional signals are not stationary; all spatial frequency components, from short- (up to 16 kbp) to long-range are found in certain regions of the chromosome but not in others (Jeong et al., 2004; Allen et al., 2006). Furthermore, different parts of the chromosome may contain different amount of spectral information. For example, nearly all spatial frequency components can be found in a region of the chromosome proximal to the origin of replication in the counter-clockwise replichore (Jeong et al., 2004). In fact, the presence of multiple frequency components, up to 128 ± 12 kbp, in this region is a characteristic feature of spatial transcriptional profiles obtained under dozens of conditions, including growth phase transitions, nutritional shifts, recoveries, antibiotic treatments, etc. (Jeong et al., 2004, 2006; Sangurdekar et al., 2006; Xiao et al., 2011). The exceptions were DNA damaging conditions causing inhibition of DNA replication, including treatment with a quinolone antibiotic, norfloxacin (Jeong et al., 2004, 2006), and thymine starvation leading to TLD (Figure 1). A study by Jeong et al. (2006) demonstrated, using mutants with conditionally defective initiation of chromosomal DNA replication, that ongoing replication is required for observing spatial patterns across scales. Both the quinolone treatment (Goss et al., 1965; Benbrook and Miller, 1986; Khodursky and Cozzarelli, 1998) and thymine starvation (Barner and Cohen, 1958; Kuong and Kuzminov, 2010) result in rapid inhibition of DNA replication, which in turn likely brings about structural changes in the chromosome that disfavor spatial pattern formation.

## State of DNA in Thymine Starved Cells

Observations of the dissolution of spatial transcriptional patterns may be confounded in part by the fact that the loss of the patterns occurs under conditions that induce DNA damage. The damaged DNA may serve as a poor template for transcription, thereby providing an almost trivial reason for the loss of the spatial correlations in transcriptional activity.

Indeed, several types of DNA damage have been observed in cells starved for thymine, some of which may culminate in the template destruction: single strand breaks in DNA (Freifelder, 1969; Hill and Fangman, 1973; Nakayama and Hanawalt, 1975); double strand breaks (Yoshinaga, 1973; Guarino et al., 2007; Kuong and Kuzminov, 2010, 2012); geographically limited degradation of *Bacillus subtilis* chromosomal DNA behind the replication fork (Ramareddy and Reiter, 1970; Reiter and Ramareddy, 1970) and on one side of the origin of replication (Regamey et al., 2000); partial unstructuring of the *E. coli* chromosome in a region around the origin of replication (Nakayama et al., 1994). It was shown that DNA from thymine starved cells is indeed susceptible to a single strand endonuclease activity *in vitro* and that thymine starvation induces transcription of the ssb gene, encoding the single strand DNA binding protein, which is consistent with an increase in the fraction of single strand DNA *in vivo* (Sangurdekar et al., 2010). Furthermore, investigations of DNA metabolism indicated that the starvation not only inhibits normal elongation of DNA replication (Maaloe and Hanawalt, 1961) but also results in a residual DNA synthesis (Kuong and Kuzminov, 2010) and small but quantifiable increase in the amount of bulk DNA (Barner and Cohen, 1958; Breitman et al., 1972; Kuong and Kuzminov, 2012), which is concomitant with the loss of thymine from DNA (Breitman et al., 1972) and is followed by contained DNA degradation (Kuong and Kuzminov, 2012).

**FIGURE 2:**
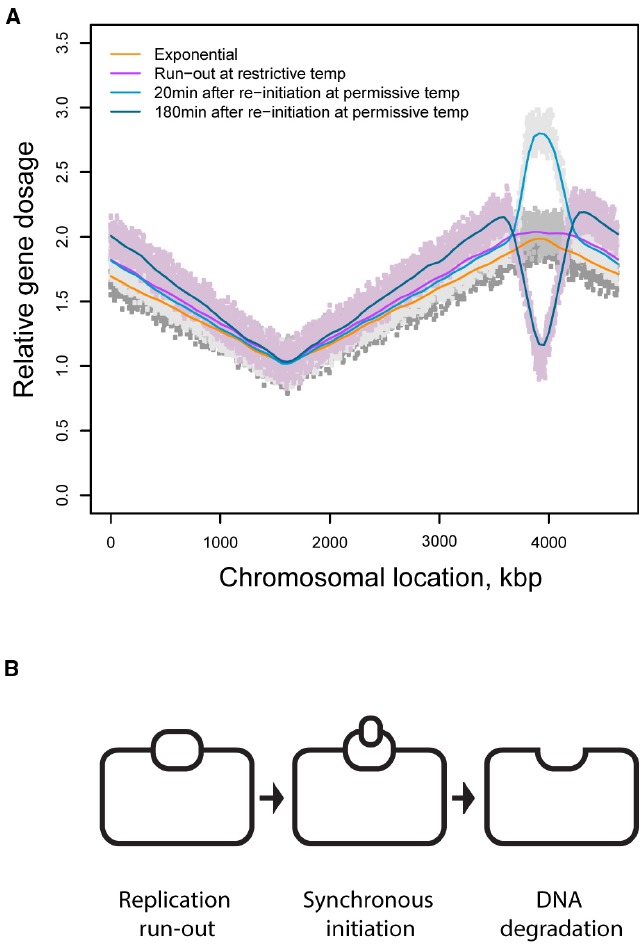
**(A)** DNA dosage profiles in chromosomes of cells synchronized for replication initiation by using a temperature sensitive replication initiation mutant (Hamann, 2013). **(B)** A model of DNA loss in thymine starved cells synchronized for initiation of DNA replication.

Thus DNA degradation appears to be the main confounding factor in interpreting the loss of spatial transcriptional patterns in the vicinity of the origin of replication. In their attempt to map single strand DNA regions and/or regions of unstructured DNA, Sangurdekar et al. used the array comparative genomic hybridization (aCGH) method (Sangurdekar et al., 2010), which was earlier adapted (Khodursky et al., 2000b) for genetic marker frequency analysis (Sueoka and Yoshikawa, 1965) with a single gene resolution. Hybridization of labeled genomic DNA to microarray probes representing nearly every single gene and intergenic sequence in the *E. coli* genome (Khodursky et al., 2003) results in a characteristic profile of average abundances of sequences along the chromosome in a population of cells. It was shown that thymine starvation triggers gradual loss of DNA in the 500 kbp region surrounding the origin of replication (Sangurdekar et al., 2010): in the course of starvation the average dosage of DNA at and in the immediate vicinity of the origin of replication is reduced to the dosage of the replication terminus, the area of the chromosome present at the lowest frequency in an exponentially growing bacterial population (Figure 2A). Comparable localized variations in gene dosage have been observed by Rosenberg and colleagues (Fonville et al., 2010) and by Kuong and Kuzminov (2012). These results are consistent with a model according to which DNA template collapses at the replication origin and its neighborhood either as a result of degradation of nascent leading and lagging DNA strands in both chromatids or from a random, relatively short patch degradation of parental and nascent DNA, also in both chromatids (Sangurdekar et al., 2010). Both interpretations imply that DNA in the region may be degenerated to a point where it can no longer be used as a template in the reaction of transcription, explaining why any transcriptional patterns in that region might also be degraded. This view however, is complicated by an observation that thymine deprived cells continue transcribing genes in the region of the lesion well into starvation and even during the killing phase of TLD (Sangurdekar et al., 2010): half-life of a representative *E. coli* mRNA is about 60 times shorter than the timeline of a typical thymine starvation experiment (Bernstein et al., 2002) and there is no indication that mRNA stability is affected under conditions of thymine starvation, and the average abundance of transcripts from the region in question changes less than 50% in the course of the starvation (Sangurdekar et al., 2010). Thus an alternative model of the formation of the origin-centric lesion must be entertained, in which at least one DNA template remains intact.

Figure 2A depicts DNA dosage variations in a population of the thymine starved cells synchronized for initiation of DNA replication using a dnaC^ts^ mutant (Hamann, 2013); a similar profile with analogous interpretation was independently obtained by Kuong and Kuzminov (2012) in an asynchronous population of starved cells. Three conclusions can be drawn from this observation. First, following thymine withdrawal from a population of cells that can no longer initiate new rounds of replication, replication continues at least in a fraction of cells for a short period of time and then stops, resulting in a characteristic plateau spanning the stretch of the chromosome that underwent residual replication. Second, upon return to the permissive temperature, starved cells can initiate new rounds of replication even though the cells were deprived of thymine and could only support limited extension of the ongoing replication rounds. Third, new rounds of replication are initiated only on one of two partially replicated sister chromatids followed by complete destruction of the newly established replication bubble. (Although the increase in DNA dosage may be interpreted as a variation in a fraction of the population, the decrease cannot be explained in fractional terms.). Such sequence of events would result in one intact DNA template (Figure 2B). Although this template is transcriptionally active, its activity is spatially disorganized.

## Concluding Remarks

Spatial regularity is one of the attributes of transcriptional activity of living genomes. The nature of spatial correlations at linear distances greater than the average size of an operon is poorly understood. The patterns somewhat coincide with the purported structural features of the chromosomal DNA and may in small part be explained by co-regulation of genes that are spaced with some periodicity on the chromosome (Kepes, 2004). The correlations are particularly sensitive to the state of DNA. Conditions that interfere with either DNA supercoiling or DNA replication result in diminishment or dissolution of the patterns, suggesting that the correlations are set, or modulated, by the moving replication fork.

Thymine starvation is one of the conditions that inhibit spatial pattern formation in the region of the chromosome adjacent to the origin of replication. However, this condition not merely inhibits DNA replication but also results in structural, copy number variations in the same region of the chromosome that loses the spatial pattern and yet does not become transcriptionally silent. It raises a formal possibility that, if spatial transcriptional correlations are the result of chromosomal DNA folding behind the replication fork, the normal folding of the chromosome in a region may also depend on the regional gene dosage or ploidy of the region. Moreover, the behind-the-fork organization of the chromosomal DNA into supercoiled loops (Postow et al., 2004) or rosettes of similar size loops (Kavenoff and Bowen, 1976) may result in a coordinated, basal transcriptional activity along the newly replicated stretch of DNA. This activity may facilitate further chromosomal folding which in turn may be required for the following round of replication initiation and nucleoid segregation. Consistent with this view are observations that global inhibition of transcription results in chromosome decondensation (Pettijohn and Hecht, 1974) and precludes initiation of DNA replication without a need for locus-specific transcription (Bates et al., 1997).

### Conflict of Interest Statement

The authors declare that the research was conducted in the absence of any commercial or financial relationships that could be construed as a potential conflict of interest.

## References

[B1] Allen T. E. Price N. D. Joyce A. R. Palsson B. O. (2006). Long-range periodic patterns in microbial genomes indicate significant multi-scale chromosomal organization. PLoS Comput. Biol. 2:e2. 10.1371/journal.pcbi.0020002 16410829 PMC1326223

[B2] Barner H. D. Cohen S. S. (1954). The induction of thymine synthesis by T2 infection of a thymine requiring mutant of *Escherichia coli*. J. Bacteriol. 68, 80–88. 13183905 10.1128/jb.68.1.80-88.1954 PMC357338

[B3] Barner H. D. Cohen S. S. (1956). The relation of growth to the lethal damage induced by ultraviolet irradiation in *Escherichia coli*. J. Bacteriol. 71, 149–157. 13295186 10.1128/jb.71.2.149-157.1956 PMC357757

[B4] Barner H. D. Cohen S. S. (1957). The isolation and properties of amino acid requiring mutants of a thymineless bacterium. J. Bacteriol. 74, 350–355. 13475248 10.1128/jb.74.3.350-355.1957 PMC314646

[B5] Barner H. D. Cohen S. S. (1958). Protein synthesis and RNA turnover in a pyrimidine-deficient bacterium. Biochim. Biophys. Acta 30, 12–20. 10.1016/0006-3002(58)90234-8 13584390

[B6] Bates D. B. Boye E. Asai T. Kogoma T. (1997). The absence of effect of gid or mioC transcription on the initiation of chromosomal replication in *Escherichia coli*. Proc. Natl. Acad. Sci. U.S.A. 94, 12497–12502. 10.1073/pnas.94.23.12497 9356478 PMC25015

[B7] Belfort M. Maley G. Pedersen-Lane J. Maley F. (1983). Primary structure of the *Escherichia coli* thyA gene and its thymidylate synthase product. Proc. Natl. Acad. Sci. U.S.A. 80, 4914–4918. 10.1073/pnas.80.16.4914 6308660 PMC384157

[B8] Benbrook D. M. Miller R. V. (1986). Effects of norfloxacin on DNA metabolism in *Pseudomonas aeruginosa*. Antimicrob. Agents Chemother. 29, 1–6. 10.1128/AAC.29.1.1 3015000 PMC180353

[B9] Bernstein J. A. Khodursky A. B. Lin P. H. Lin-Chao S. Cohen S. N. (2002). Global analysis of mRNA decay and abundance in *Escherichia coli* at single-gene resolution using two-color fluorescent DNA microarrays. Proc. Natl. Acad. Sci. U.S.A. 99, 9697–9702. 10.1073/pnas.112318199 12119387 PMC124983

[B10] Breitman T. R. Maury P. B. Toal J. N. (1972). Loss of deoxyribonucleic acid-thymine during thymine starvation of *Escherichia coli*. J. Bacteriol. 112, 646–648. 4562414 10.1128/jb.112.1.646-648.1972 PMC251463

[B11] Burton Z. F. Gross C. A. Watanabe K. K. Burgess R. R. (1983). The operon that encodes the sigma subunit of RNA polymerase also encodes ribosomal protein S21 and DNA primase in *E. coli* K12. Cell 32, 335–349. 10.1016/0092-8674(83)90453-1 6186393

[B12] Cagliero C. Zhou Y. N. Jin D. J. (2014). Spatial organization of transcription machinery and its segregation from the replisome in fast-growing bacterial cells. Nucleic Acids Res. 42, 13696–13705. 10.1093/nar/gku1103 25416798 PMC4267616

[B13] Casaregola S. D’ari R. Huisman O. (1982). Role of DNA replication in the induction and turn-off of the SOS response in *Escherichia coli*. Mol. Gen. Genet. 185, 440–444. 10.1007/BF00334136 6808321

[B14] Chandler M. G. Pritchard R. H. (1975). The effect of gene concentration and relative gene dosage on gene output in *Escherichia coli*. Mol. Gen. Genet. 138, 127–141. 10.1007/BF02428117 1105148

[B15] Chiaramello A. E. Zyskind J. W. (1990). Coupling of DNA replication to growth rate in *Escherichia coli*: a possible role for guanosine tetraphosphate. J. Bacteriol. 172, 2013–2019. 1690706 10.1128/jb.172.4.2013-2019.1990 PMC208699

[B16] Cohen S. S. Barner H. D. (1954). Studies on Unbalanced Growth in *Escherichia Coli*. Proc. Natl. Acad. Sci. U.S.A. 40, 885–893. 10.1073/pnas.40.10.885 16589586 PMC534191

[B17] Courcelle J. Khodursky A. Peter B. Brown P. O. Hanawalt P. C. (2001). Comparative gene expression profiles following UV exposure in wild-type and SOS-deficient *Escherichia coli*. Genetics 158, 41–64. 11333217 10.1093/genetics/158.1.41 PMC1461638

[B18] Couturier E. Rocha E. P. (2006). Replication-associated gene dosage effects shape the genomes of fast-growing bacteria but only for transcription and translation genes. Mol. Microbiol. 59, 1506–1518. 10.1111/j.1365-2958.2006.05046.x 16468991

[B19] Crooke E. Hwang D. S. Skarstad K. Thony B. Kornberg A. (1991). *E. coli* minichromosome replication: regulation of initiation at *oriC*. Res. Microbiol. 142, 127–130. 10.1016/0923-2508(91)90019-7 1925009

[B20] Denapoli J. Tehranchi A. K. Wang J. D. (2013). Dose-dependent reduction of replication elongation rate by (p)ppGpp in *Escherichia coli* and *Bacillus subtilis*. Mol. Microbiol. 88, 93–104. 10.1111/mmi.12172 23461544 PMC3640871

[B21] Dorman C. J. (2006). DNA supercoiling and bacterial gene expression. Sci. Prog. 89, 151–166. 10.3184/003685006783238317 17338437 PMC10368349

[B22] Eisen M. B. Spellman P. T. Brown P. O. Botstein D. (1998). Cluster analysis and display of genome-wide expression patterns. Proc. Natl. Acad. Sci. U.S.A. 95, 14863–14868. 10.1073/pnas.95.25.14863 9843981 PMC24541

[B23] Felipe-Abrio I. Lafuente-Barquero J. Garcia-Rubio M. L. Aguilera A. (2014). RNA polymerase II contributes to preventing transcription-mediated replication fork stalls. EMBO J. 34, 236–250. 10.15252/embj.201488544 25452497 PMC4337070

[B24] Fonville N. C. Bates D. Hastings P. J. Hanawalt P. C. Rosenberg S. M. (2010). Role of RecA and the SOS response in thymineless death in *Escherichia coli*. PLoS Genet. 6:e1000865. 10.1371/journal.pgen.1000865 20221259 PMC2832678

[B25] Freifelder D. (1969). Single-strand breaks in bacterial DNA associated with thymine starvation. J. Mol. Biol. 45, 1–7. 10.1016/0022-2836(69)90205-8 4898842

[B26] French S. (1992). Consequences of replication fork movement through transcription units *in vivo*. Science 258, 1362–1365. 10.1126/science.1455232 1455232

[B27] Gallant J. Suskind S. R. (1962). Ribonucleic acid synthesis and thymineless death. Biochim. Biophys. Acta 55, 627–638 10.1016/0006-3002(62)90841-7 13896401

[B28] Goss W. A. Deitz W. H. Cook T. M. (1965). Mechanism of action of nalidixic acid on *Escherichia Coli*. ii. inhibition of deoxyribonucleic acid synthesis. J. Bacteriol. 89, 1068–1074. 14276097 10.1128/jb.89.4.1068-1074.1965 PMC277597

[B29] Guarino E. Salguero I. Jimenez-Sanchez A. Guzman E. C. (2007). Double-strand break generation under deoxyribonucleotide starvation in *Escherichia coli*. J. Bacteriol. 189, 5782–5786. 10.1128/JB.00411-07 17526701 PMC1951825

[B30] Gutierrez-Rios R. M. Rosenblueth D. A. Loza J. A. Huerta A. M. Glasner J. D. Blattner F. R. (2003). Regulatory network of *Escherichia coli*: consistency between literature knowledge and microarray profiles. Genome Res. 13, 2435–2443. 10.1101/gr.1387003 14597655 PMC403762

[B31] Hamann B. L. (2013). Elucidating the Mechanism of Thymineless Death in Escherichia coli. Ph.D. thesis, University of Minnesota, Minnesota.

[B32] Hanawalt P. C. (1963). Involvement of synthesis of RNA in thymineless death. Nature 198, 286. 10.1038/198286a0 13952474

[B33] Hill W. E. Fangman W. L. (1973). Single-strand breaks in deoxyribonucleic acid and viability loss during deoxyribonucleic acid synthesis inhibition in *Escherichia coli*. J. Bacteriol. 116, 1329–1335. 4584811 10.1128/jb.116.3.1329-1335.1973 PMC246491

[B34] Jeong K. S. Ahn J. Khodursky A. B. (2004). Spatial patterns of transcriptional activity in the chromosome of *Escherichia coli*. Genome Biol. 5, R86. 10.1186/gb-2004-5-11-r86 15535862 PMC545777

[B35] Jeong K. S. Xie Y. Hiasa H. Khodursky A. B. (2006). Analysis of pleiotropic transcriptional profiles: a case study of DNA gyrase inhibition. PLoS Genet. 2:e152. 10.1371/journal.pgen.0020152 17009874 PMC1584274

[B36] Jin D. J. Cagliero C. Zhou Y. N. (2012). Growth rate regulation in *Escherichia coli*. FEMS Microbiol. Rev. 36, 269–287. 10.1111/j.1574-6976.2011.00279.x 21569058 PMC3478676

[B37] Kaguni J. M. (2006). DnaA: controlling the initiation of bacterial DNA replication and more. Annu. Rev. Microbiol. 60, 351–375. 10.1146/annurev.micro.60.080805.142111 16753031

[B38] Kavenoff R. Bowen B. C. (1976). Electron microscopy of membrane-free folded chromosomes from *Escherichia coli*. Chromosoma 59, 89–101. 10.1007/BF00328479 795620

[B39] Kepes F. (2004). Periodic transcriptional organization of the *E.coli* genome. J. Mol. Biol. 340, 957–964. 10.1016/j.jmb.2004.05.039 15236959

[B40] Khodursky A. B. Bernstein J. A. Peter B. J. Rhodius V. Wendisch V. F. Zimmer D. P. (2003). *Escherichia coli* spotted double-strand DNA microarrays: RNA extraction, labeling, hybridization, quality control, and data management. Methods Mol. Biol. 224, 61–78. 10.1385/1-59259-364-X:61 12710666

[B41] Khodursky A. B. Cozzarelli N. R. (1998). The mechanism of inhibition of topoisomerase IV by quinolone antibacterials. J. Biol. Chem. 273, 27668–27677. 10.1074/jbc.273.42.27668 9765303

[B42] Khodursky A. B. Peter B. J. Cozzarelli N. R. Botstein D. Brown P. O. Yanofsky C. (2000a). DNA microarray analysis of gene expression in response to physiological and genetic changes that affect tryptophan metabolism in *Escherichia coli*. Proc. Natl. Acad. Sci. U.S.A. 97, 12170–12175. 10.1073/pnas.220414297 11027315 PMC17313

[B43] Khodursky A. B. Peter B. J. Schmid M. B. Derisi J. Botstein D. Brown P. O. (2000b). Analysis of topoisomerase function in bacterial replication fork movement: use of DNA microarrays. Proc. Natl. Acad. Sci. U.S.A. 97, 9419–9424. 10.1073/pnas.97.17.9419 10944214 PMC16879

[B44] Kowalski D. Eddy M. J. (1989). The DNA unwinding element: a novel, *cis*-acting component that facilitates opening of the *Escherichia coli* replication origin. EMBO J. 8, 4335–4344. 2556269 10.1002/j.1460-2075.1989.tb08620.x PMC401646

[B45] Kreuzer K. N. (2013). DNA damage responses in prokaryotes: regulating gene expression, modulating growth patterns, and manipulating replication forks. Cold Spring Harb. Perspect. Biol. 5, a012674. 10.1101/cshperspect.a012674 24097899 PMC3809575

[B46] Kuong K. J. Kuzminov A. (2010). Stalled replication fork repair and misrepair during thymineless death in *Escherichia coli*. Genes Cells 15, 619–634. 10.1111/j.1365-2443.2010.01405.x 20465561 PMC3965187

[B47] Kuong K. J. Kuzminov A. (2012). Disintegration of nascent replication bubbles during thymine starvation triggers RecA- and RecBCD-dependent replication origin destruction. J. Biol. Chem. 287, 23958–23970. 10.1074/jbc.M112.359687 22621921 PMC3390671

[B48] Lark K. G. (1972). Evidence for the direct involvement of RNA in the initiation of DNA replication in *Escherichia coli* 15T. J. Mol. Biol. 64, 47–60. 10.1016/0022-2836(72)90320-8 4552485

[B49] Le T. B. Imakaev M. V. Mirny L. A. Laub M. T. (2013). High-resolution mapping of the spatial organization of a bacterial chromosome. Science 342, 731–734. 10.1126/science.1242059 24158908 PMC3927313

[B50] Levine A. Vannier F. Dehbi M. Henckes G. Seror S. J. (1991). The stringent response blocks DNA replication outside the ori region in *Bacillus subtilis* and at the origin in *Escherichia coli*. J. Mol. Biol. 219, 605–613. 10.1016/0022-2836(91)90657-R 1905358

[B51] Lieberman-Aiden E. Van Berkum N. L. Williams L. Imakaev M. Ragoczy T. Telling A. (2009). Comprehensive mapping of long-range interactions reveals folding principles of the human genome. Science 326, 289–293. 10.1126/science.1181369 19815776 PMC2858594

[B52] Liu B. Alberts B. M. (1995). Head-on collision between a DNA replication apparatus and RNA polymerase transcription complex. Science 267, 1131–1137. 10.1126/science.7855590 7855590

[B53] Maaloe O. Hanawalt P. C. (1961). Thymine deficiency and the normal DNA replication cycle. I. J. Mol. Biol. 3, 144–155. 10.1016/S0022-2836(61)80041-7 13764647

[B54] Martin C. M. Viguera E. Guzman E. C. (2014). Rifampicin suppresses thymineless death by blocking the transcription-dependent step of chromosome initiation. DNA Repair (Amst) 18, 10–17. 10.1016/j.dnarep.2014.03.004 24742961

[B55] McFall E. Magasanik B. (1960). Thymine starvation and enzyme synthesis. Biochim. Biophys. Acta 45, 610–612 10.1016/0006-3002(60)91505-5 13773908

[B56] Merrikh H. Machon C. Grainger W. H. Grossman A. D. Soultanas P. (2011). Co-directional replication-transcription conflicts lead to replication restart. Nature 470, 554–557. 10.1038/nature09758 21350489 PMC3059490

[B57] Merrikh H. Zhang Y. Grossman A. D. Wang J. D. (2012). Replication-transcription conflicts in bacteria. Nat. Rev. Microbiol. 10, 449–458. 10.1038/nrmicro2800 22669220 PMC3467967

[B58] Messer W. (1972). Initiation of deoxyribonucleic acid replication in *Escherichia coli* B-r: chronology of events and transcriptional control of initiation. J. Bacteriol. 112, 7–12. 4562418 10.1128/jb.112.1.7-12.1972 PMC251374

[B59] Morganroth P. A. Hanawalt P. C. (2006). Role of DNA replication and repair in thymineless death in *Escherichia coli*. J. Bacteriol. 188, 5286–5288. 10.1128/JB.00543-06 16816201 PMC1539979

[B60] Nakayama H. Hanawalt P. (1975). Sedimentation analysis of deoxyribonucleic acid from thymine-starved *Escherichia coli*. J. Bacteriol. 121, 537–547. 1090581 10.1128/jb.121.2.537-547.1975 PMC245964

[B61] Nakayama K. Kusano K. Irino N. Nakayama H. (1994). Thymine starvation-induced structural changes in *Escherichia coli* DNA. Detection by pulsed field gel electrophoresis and evidence for involvement of homologous recombination. J. Mol. Biol. 243, 611–620. 10.1016/0022-2836(94)90036-1 7966286

[B62] Olliver A. Saggioro C. Herrick J. Sclavi B. (2010). DnaA-ATP acts as a molecular switch to control levels of ribonucleotide reductase expression in *Escherichia coli*. Mol. Microbiol. 76, 1555–1571. 10.1111/j.1365-2958.2010.07185.x 20487274

[B63] Peter B. J. Arsuaga J. Breier A. M. Khodursky A. B. Brown P. O. Cozzarelli N. R. (2004). Genomic transcriptional response to loss of chromosomal supercoiling in *Escherichia coli*. Genome Biol. 5, R87. 10.1186/gb-2004-5-11-r87 15535863 PMC545778

[B64] Pettijohn D. E. Hecht R. (1974). RNA molecules bound to the folded bacterial genome stabilize DNA folds and segregate domains of supercoiling. Cold Spring Harb. Symp. Quant. Biol. 38, 31–41. 10.1101/SQB.1974.038.01.006 4598638

[B65] Postow L. Hardy C. D. Arsuaga J. Cozzarelli N. R. (2004). Topological domain structure of the *Escherichia coli* chromosome. Genes Dev. 18, 1766–1779. 10.1101/gad.1207504 15256503 PMC478196

[B66] Ramareddy G. Reiter H. (1970). Sequential loss of loci in thymine-starved *Bacillus subtilis* 168 cells. Evidence for a circular chromosome. J. Mol. Biol. 50, 525–532. 10.1016/0022-2836(70)90209-3 4991006

[B67] Regamey A. Harry E. J. Wake R. G. (2000). Mid-cell Z ring assembly in the absence of entry into the elongation phase of the round of replication in bacteria: co-ordinating chromosome replication with cell division. Mol. Microbiol. 38, 423–434. 10.1046/j.1365-2958.2000.02130.x 11069667

[B68] Reichard P. (2010). Ribonucleotide reductases: substrate specificity by allostery. Biochem. Biophys. Res. Commun. 396, 19–23. 10.1016/j.bbrc.2010.02.108 20494104

[B69] Reiter H. Ramareddy G. (1970). Loss of DNA behind the growing point of thymine-starved *Bacillus subtilis* 168. J. Mol. Biol. 50, 533–548. 10.1016/0022-2836(70)90210-X 4991007

[B70] Rolfe R. (1967). On the mechanism of thymineless death in *Bacillus subtilis*. Proc. Natl. Acad. Sci. U.S.A. 57, 114–121. 10.1073/pnas.57.1.114 4963251 PMC335472

[B71] Rovinskiy N. Agbleke A. A. Chesnokova O. Pang Z. Higgins N. P. (2012). Rates of gyrase supercoiling and transcription elongation control supercoil density in a bacterial chromosome. PLoS Genet. 8:e1002845. 10.1371/journal.pgen.1002845 22916023 PMC3420936

[B72] Samuel G. Reeves P. (2003). Biosynthesis of O-antigens: genes and pathways involved in nucleotide sugar precursor synthesis and O-antigen assembly. Carbohydr. Res. 338, 2503–2519. 10.1016/j.carres.2003.07.009 14670712

[B73] Sangurdekar D. P. Hamann B. L. Smirnov D. Srienc F. Hanawalt P. C. Khodursky A. B. (2010). Thymineless death is associated with loss of essential genetic information from the replication origin. Mol. Microbiol. 75, 1455–1467. 10.1111/j.1365-2958.2010.07072.x 20132444

[B74] Sangurdekar D. P. Srienc F. Khodursky A. B. (2006). A classification based framework for quantitative description of large-scale microarray data. Genome Biol. 7, R32. 10.1186/gb-2006-7-4-r32 16626502 PMC1557986

[B75] Sangurdekar D. P. Zhang Z. Khodursky A. B. (2011). The association of DNA damage response and nucleotide level modulation with the antibacterial mechanism of the anti-folate drug trimethoprim. BMC Genomics 12:583. 10.1186/1471-2164-12-583 22122981 PMC3258297

[B76] Schena M. Shalon D. Davis R. W. Brown P. O. (1995). Quantitative monitoring of gene expression patterns with a complementary DNA microarray. Science 270, 467–470. 10.1126/science.270.5235.467 7569999

[B77] Schmid M. B. Roth J. R. (1987). Gene location affects expression level in *Salmonella typhimurium*. J. Bacteriol. 169, 2872–2875. 3294809 10.1128/jb.169.6.2872-2875.1987 PMC212203

[B78] Simmons L. A. Foti J. J. Cohen S. E. Walker G. C. (2008). The SOS regulatory network. Ecosal Plus 2008, 1–48. 10.1128/ecosalplus.5.4.3 26443738

[B79] Slager J. Kjos M. Attaiech L. Veening J. W. (2014). Antibiotic-induced replication stress triggers bacterial competence by increasing gene dosage near the origin. Cell 157, 395–406. 10.1016/j.cell.2014.01.068 24725406

[B80] Sobetzko P. Travers A. Muskhelishvili G. (2012). Gene order and chromosome dynamics coordinate spatiotemporal gene expression during the bacterial growth cycle. Proc. Natl. Acad. Sci. U.S.A. 109, E42–E50. 10.1073/pnas.1108229109 22184251 PMC3258614

[B81] Sueoka N. Yoshikawa H. (1965). The chromosome of *Bacillus subtilis*. I. Theory of marker frequency analysis. Genetics 52, 747–757. 4953222 10.1093/genetics/52.4.747 PMC1210937

[B82] Tamayo P. Slonim D. Mesirov J. Zhu Q. Kitareewan S. Dmitrovsky E. (1999). Interpreting patterns of gene expression with self-organizing maps: methods and application to hematopoietic differentiation. Proc. Natl. Acad. Sci. U.S.A. 96, 2907–2912. 10.1073/pnas.96.6.2907 10077610 PMC15868

[B83] Torrence C. Compo G. (1998). A practical guide to wavelet analysis. Bull. Amer. Met. Soc. 79, 61–78 10.1175/1520-0477(1998)079<0061:APGTWA>2.0.CO;2

[B84] van Helden J. Andre B. Collado-Vides J. (1998). Extracting regulatory sites from the upstream region of yeast genes by computational analysis of oligonucleotide frequencies. J. Mol. Biol. 281, 827–842. 10.1006/jmbi.1998.1947 9719638

[B85] Versalovic J. Koeuth T. Britton R. Geszvain K. Lupski J. R. (1993). Conservation and evolution of the rpsU-dnaG-rpoD macromolecular synthesis operon in bacteria. Mol. Microbiol. 8, 343–355. 10.1111/j.1365-2958.1993.tb01578.x 8316085

[B86] von Freiesleben U. Rasmussen K. V. (1992). The level of supercoiling affects the regulation of DNA replication in *Escherichia coli*. Res. Microbiol. 143, 655–663. 10.1016/0923-2508(92)90060-2 1488550

[B87] Xiao G. Wang X. Khodursky A. B. (2011). Modeling three-dimensional chromosome structures using gene expression data. J. Am. Stat. Assoc. 106, 61–72. 10.1198/jasa.2010.ap09504 21760653 PMC3134274

[B88] Yoshinaga K. (1973). Double-strand scission of DNA involved in thymineless death of *Escherichia coli* 15 TAU. Biochim. Biophys. Acta 294, 204–213 10.1016/0005-2787(73)90293-1 4575960

